# Lay Social Resources for Support of Adherence to Antiretroviral Prophylaxis for HIV Prevention Among Serodiscordant Couples in sub-Saharan Africa: A Qualitative Study

**DOI:** 10.1007/s10461-014-0899-4

**Published:** 2014-10-01

**Authors:** Norma C. Ware, Emily E. Pisarski, Jessica E. Haberer, Monique A. Wyatt, Elioda Tumwesigye, Jared M. Baeten, Connie L. Celum, David R. Bangsberg

**Affiliations:** 1 Department of Global Health and Social Medicine, Harvard Medical School, 641 Huntington Ave., Boston, MA USA; 2 Department of Psychiatry, Harvard Medical School, Boston, MA USA; 3 Center for Global Health, Massachusetts General Hospital, Boston, MA USA; 4 Department of Medicine, Harvard Medical School, Boston, MA USA; 5 Kabwohe Clinical Research Center, Kabwohe, Uganda; 6 Department of Global Health, University of Washington, Seattle WA, USA; 7 Department of Medicine, University of Washington, Seattle, WA USA; 8 Department of Epidemiology, University of Washington, Seattle, WA USA; 9 Department of Medicine, Mbarara University of Science and Technology, Mbarara, Uganda

**Keywords:** HIV Prevention, PrEP, Adherence, Serodiscordant Couples, Sub-Saharan Africa, HIV Health Care Delivery in Africa

## Abstract

Effectiveness of antiretroviral pre-exposure prophylaxis (PrEP) for HIV prevention will require high adherence. Using qualitative data, this paper identifies potential lay social resources for support of PrEP adherence by HIV serodiscordant couples in Uganda, laying the groundwork for incorporation of these resources into adherence support initiatives as part of implementation. The qualitative analysis characterizes support for PrEP adherence provided by HIV-infected spouses, children, extended family members, and the larger community. Results suggest social resources for support of PrEP adherence in Africa are plentiful outside formal health care settings and health systems and that couples will readily use them. The same shortage of health professionals that impeded scale-up of antiretroviral treatment for HIV/AIDS in Africa promises to challenge delivery of PrEP. Building on the treatment scale-up experience, implementers can address this challenge by examining the value of lay social resources for adherence support in developing strategies for delivery of PrEP.

## Resumen

La efectividad de la profilaxis de pre-exposición (PrPE) para la prevención de VIH requerirá alta adherencia. Utilizando información cualitativa, este articulo identifica potenciales recursos en la sociedad civil para apoyar la adherencia a PrPE por parejas serodiscordantes en Uganda, estableciendo las bases para incorporar estos recursos en las iniciativas de apoyo de adherencia como parte de la implementación. El análisis cualitativo caracteriza el apoyo para adherencia a PrPE proporcionado por parejas infectadas por VIH, niños, otros miembros de la familia y la comunidad en general. Los resultados sugieren que los recursos sociales de apoyo para adherencia a PrPE en África son abundantes fuera de las los establecimientos de salud formales y del sistema de salud y que las parejas los utilizarían fácilmente. La misma escasez de profesionales de salud que impidió la implementación a gran escala del tratamiento anti-retroviral para VIH/SIDA en África promete desafiar la entrega de PrPE. Construyendo sobre la experiencia de la implementación a gran escala del tratamiento, implementadores pueden abordar este desafío examinando el valor de los recursos de la sociedad civil para apoyar la adherencia en el desarrollo de estrategias para la entrega de PrPE.

## Introduction

Plans and preparations for implementing HIV prevention strategies using antiretrovirals (ARVs) are being debated internationally. Evidence of efficacy of oral tenofovir disoproxil fumarate (TDF) and coformulated emtricitabine/tenofovir disoproxil fumarate (FTC/TDF) as pre-exposure prophylaxis (PrEP) has been demonstrated for HIV-uninfected individuals [1–4] and approved by the US Food and Drug Administration [5]. Mathematical modeling studies examining PrEP impact in different populations highlight the importance of risk level and adherence in determining cost effectiveness [6–11]. Guidance on implementation approaches is emerging [12, 13]. A number of demonstration projects evaluating delivery of PrEP are underway in the US [14–20], and several more are taking place and/or being planned in sub-Saharan Africa [21–23].

HIV serodiscordant couples represent the priority population for implementation of ARV-based HIV prevention in Africa. Half of HIV-infected persons in marital or cohabiting relationships have partners who are not infected [24–26]. Rates of HIV transmission in serodiscordant couples are high, ranging from 2 % to almost 12 % [27–30]. Serodiscordant couples can be targeted for prevention efforts through promotion of couples HIV counseling and testing [31].

International scale-up of antiretroviral therapy (ART) for treatment of HIV-infection is considered a resounding public health success [32–36]. The success of the scale-up initiative has been achieved despite significant implementation challenges, prominent among them being shortages of personnel qualified to deliver ART and provide clinical follow-up care [37]. The human resources challenge has been addressed in part through steadily increasing involvement of laypersons in activities of care. Integrating ARVs for prevention into HIV-related health services in Africa will place additional responsibility on already overburdened health systems.

Effectiveness of PrEP depends largely on adherence, as indicated by the association between drug levels as an objective adherence measure and efficacy in the four PrEP trials in which efficacy has been demonstrated [1–4, 38–45]. Adherence to ARVs for HIV treatment in Africa has proven excellent [46–52], despite major structural barriers to accessing and persisting in care [53–59]. Adherence to ARVs for HIV prevention and the impact of potential adherence barriers outside of clinical trials are not yet well understood.

Excellent adherence to ARVs as HIV treatment in Africa reflects a number of contributing factors. Great emphasis has been placed on adherence as part of routine clinical follow-up care. Many clinics have professional staff (e.g., nurses, counselors) dedicated solely to providing adherence education and support. Clinics have also drawn liberally upon non-professional, community-based resources to support patients’ ART adherence efforts. For example, having a treatment partner is a condition of starting ART in many treatment programs. Treatment partners are laypersons who are charged with helping HIV-infected persons on ART adhere to daily dosing. A number of experimental studies have now documented the impact of treatment partners on ART adherence rates [60–62] and clinical outcomes [63].

“Peers” (“persons living with HIV/AIDS”) are also increasingly being recognized in Africa as a non-professional resource with important contributions to make to adherence. The roles of peers have steadily expanded from education and support to include activities of care. As ART scale-up took hold, peers were relied upon first to provide emotional support and “positive role modeling”—helping patients to accept an HIV diagnosis and “live well” with HIV/AIDS [63–65]. Over time, increases in the numbers of persons receiving ART and acceptance of task-shifting [66] have led to involvement of peers in ART drug resupply, adherence monitoring, and routine clinical follow-up [67–72]. Peer relationships and networks also perform a social integration function, helping to de-stigmatize HIV/AIDS and re-establish the standing of infected individuals in local communities [67, 73].

Whether excellent ARV adherence will be achieved in the prevention context—where the perceived need for “medicine” may be less urgent and the concept of “drugs for prevention” less familiar—is an important and very real question. The importance of adherence underscores the need to understand how individuals will cope with taking ARVs to prevent HIV infection, and how their efforts to adhere can best be supported.

The adherence successes of ART scale-up in Africa contain important lessons for replicating these successes as ARVs become available for use in prevention. One such lesson is that family members, friends and peers of persons taking ARVs are both interested in and capable of effective adherence support for HIV treatment. Understanding this, it makes sense to ask whether similar resources may be available in the prevention context. We begin to address this question by characterizing social influences on adherence observed in the Partners PrEP Study, a randomized clinical trial of daily oral PrEP for HIV prevention among HIV serodiscordant couples in East Africa.

## Methods

### Ethical Approval

This study was approved by the Partners Health Care Human Research Committee, Boston, MA; the Human Subjects Division of the University of Washington, Seattle, WA; and the Uganda Council on Science and Technology, Kampala, Uganda. Written consent was obtained from all participants.

### Study Sample

**Fig. 1:**
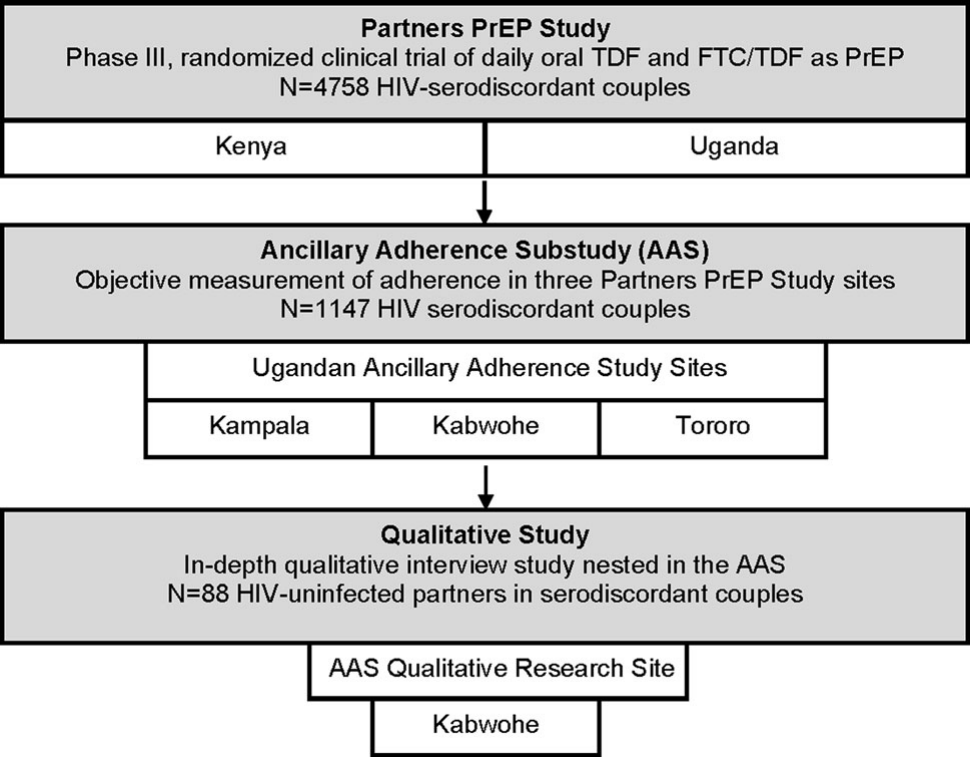
Qualitative research in relation to the ancillary adherence substudy and Partners PrEP Study

This analysis is based on data collected through in-depth qualitative interviews with 88 adults who were HIV-uninfected partners in Ugandan HIV-serodiscordant couples. Qualitative interviewees were part of an ancillary adherence substudy [44] that used validated objective measures [the electronic medication event monitoring system (MEMS), and unannounced home-based pill counts (UPCs)] to assess adherence to study product in 1,147 participants in the Partners PrEP Study from November 2009 through December 2012. The Partners PrEP Study was a multi-site, double-blind, randomized, placebo-controlled phase-III trial evaluating the safety and efficacy of daily oral TDF and FTC/TDF as PrEP to prevent HIV acquisition (ClinicalTrials.gov NCT00557245) [1]. In the adherence substudy, adherence assessment data were collected at three Partners PrEP Study sites (Kabwohe, Kampala, Tororo) in urban and rural Uganda. MEMS recorded daily adherence. Unannounced pill counts were conducted monthly for the first 6 months of study participation and quarterly thereafter. Qualitative data were collected at the adherence substudy site in Kabwohe, in rural southwest Uganda (See Fig. 1).

### Sampling Strategy

A purposeful sampling strategy was used to identify qualitative study participants. Purposeful sampling lays the groundwork for in-depth qualitative analysis by systematically representing a variety of perspectives on an object of study [74]. “Low” and “perfect” adherers to Partners PrEP Study product (TDF, FTC/TDF as PrEP, or placebo) were purposefully sampled to form participant groups with contrasting adherence experiences. Low and perfect adherence estimates were based on data from monthly or quarterly UPCs carried out as part of the adherence substudy. Low adherers were individuals whose UPC adherence dropped to 80 % or below. Perfect adherers were individuals with UPC adherence rates of 100 %. Understanding low adherence was a priority; thus all adherence substudy participants with a UPC adherence rate of </=80% were included in the qualitative study. Perfect adherers were selected from adherence substudy participants with 100 % adherence. Fifty-eight low adherers and 30 perfect adherers were included from a total of 367 ancillary adherence substudy participants at the Kabwohe site.

### Recruitment

Recruitment for the qualitative study took place over 27 months, from April 2010 to July 2012. Efficacy of PrEP for HIV prevention was demonstrated and the Partners PrEP Study unblinded in July, 2011, meaning that participants entering the qualitative study after that time had been informed they were receiving PrEP (rather than placebo) and that PrEP was efficacious [1]. Approximately half (47 %, N = 41) of study interviews took place after unblinding. Individuals identified as potential participants were approached during routine Partners PrEP Study clinical follow-up visits. They received an explanation of the research and an invitation to participate. All of the 88 individuals who were invited agreed to take part. Following recruitment, low adherers were offered an adherence support intervention as part of the adherence substudy [75]. The intervention took place after qualitative data collection was complete.

### Data Collection

Qualitative interviews were carried out by Ugandan research assistants (RAs) trained in qualitative research methodology. They took place in private locations—in patients’ homes, at the research offices, or near the clinic—where conversations could not be overheard. Conversations were audio-recorded with permission and typically lasted about an hour. A qualitative interview guide was used to address the following topics with each participant: (1) experiences of taking study pills, (2) descriptions and explanations of particular missed doses, (3) accounts of adherence lapses (when more than a single dose was missed), (4) strategies for sustaining adherence, and (5) impact of serodiscordance on the partnered relationship. Neutral probes were used to elaborate responses and elicit illustrative examples. Following each interview, RAs generated a complete transcript in English from local language audio recordings. Transcripts were reviewed for quality (detail, grammar, and style). The transcripts constitute the qualitative data analyzed for this report.

### Data Analysis

The starting point for this qualitative analysis was the explanation of PrEP adherence arrived at through our initial examination of these data, in which we identified the desire to preserve the partnered relationship as an important social motive for adherence to study product or placebo by HIV-uninfected individuals in the Partners PrEP Study [76]. Our goal was to build upon these initial findings through a broader examination of social resources for supporting PrEP adherence in the HIV prevention context. We therefore used an iterative process to re-analyze the data for evidence of other types of social influences that appeared to impact adherence.

The first and second authors reviewed the data for content illuminating the roles or potential roles of laypersons in PrEP adherence [77]. The relevance and meaning of the content was discussed. On the basis of the discussion, relevant content was organized to form the descriptive categories presented here. The categories were then illustrated using excerpts from the data.

In addition to the qualitative analysis, we compiled descriptive quantitative data on personal characteristics of the 88 qualitative participants and summarized rates of adherence.

## Results

Results of analyses are presented in the following order. Quantitative data reporting personal characteristics and adherence rates of the sample appear first, as background. These are followed by categories describing lay social resources for adherence support, the primary focus of this analysis.

### Personal Characteristics of Qualitative Study Participants

Forty-eight men (54.5 %) and 40 women (45.5 %) made up the qualitative study sample. Median age for the group was 34.5 years (IQR 29.8–39.3 years). All participants described themselves as married and living with their partners at the time of enrollment. “Marriage,” as the term is used locally, refers to unions that may or may not have been sanctioned by a religious or civil authority. Median duration of the partnered relationship was 7.6 years (IQR 4.1–16.2 years). Most (84.1 %) participants had children with their partners; median number of children was 2. Median adherence was 90 % (IQR 83.7–95.5 %) for low adherers and 100 % (IQR 99.6–100.3 %) for perfect adherers by UPC for the duration of their participation in the ancillary adherence substudy.

### Qualitative Results: Lay Social Resources for Support of PrEP Adherence

Qualitative analysis identified a number of potential lay social resources for support of PrEP adherence. These are detailed below under the headings: (1) The partnered relationship as adherence support, (2) Adherence as a “Family Affair,” and (3) Relationship support as adherence support for serodiscordant couples.

#### The Partnered Relationship as Adherence Support

Relationship partners provided direct adherence support for HIV-uninfected individuals taking study product or placebo. Support ranged from simple encouragement to “keep taking your pills,” to presenting pills and accompanying water and/or food for easy dosing at the scheduled time, to running after a departing spouse with a forgotten pill bottle in hand. Often, support involved sharing adherence responsibility by offering reminders at dosing times. Couples in which the HIV-infected partner was also taking ART or cotrimoxazole as prophylaxis often took their pills together. The following examples from interview transcripts illustrate the ways in which partners provided adherence support:

- Interviewer: “How does your partner assist you in swallowing your medicine?”
- Respondent: “Well, there are times when she reminds me. We can be there conversing and then she checks on the clock and tells me that it’s time for medicine. Then, she brings it to me and I swallow.”

Male, Low Adherer, Age 19

- Interviewer: “How did you then decide to resume taking the drugs?”
- Respondent: “When I came back home, I started again taking my drugs. This was after asking my husband what I should do, because I had feared the drugs. He tried to explain to me because for him he understood better what we had been told at the clinic. He assured me that the drugs had no problem and encouraged me to resume and I did so.”

Female, Low Adherer, Age 29

He [husband] really helps me a lot. ….He tries to remind me and he keeps encouraging me to swallow my medicine well. He tells me never to miss even for a day. If he suspects I don’t have money for transport [to clinical follow-up visits] he will meet me on the way [to the clinic] and give it to me. I have never lacked transport, not even for a single day. If he doesn’t have money, he will borrow from friends at work and bring it to me.

Female, Perfect Adherer, Age 27

- Interviewer: “How does your partner help you in swallowing your drugs?”
- Respondent: “She helps me a lot and I also help her a lot because every night she is also swallowing her drugs.”
- Interviewer: “How does she help you?”
- Respondent: “The way she helps me [is], we agreed on the time when we should be having supper, and we also agreed that by the time everyone swallows their drugs, they should have eaten supper first. So for us by 8.20 p.m. we are always through with supper. After supper, we reach for our drugs and swallow them.”

Male, Perfect Adherer, Age 44

#### Adherence as Family Affair

Partners were not the only family members providing adherence support. Children were also actively involved. Interviewee reports consistently depicted children as eager to contribute and conscientious in reminding their parents to take their pills at dosing times. Even young children proved themselves to be reliable “reminders”:

- Interviewer: “How do you remember to take your pills?”
- Respondent: “I told my child. When it clocks 7:00 p.m., he comes and tells me to swallow my medicine.”
- Interviewer: “How old is he?”
- Respondent: “He is six years.”
- Interviewer: “How does he tell the time?”
- Respondent: “I told him that I am supposed to swallow this medicine at 7:00 p.m., so he should remind me in case I forget. We don’t have a watch but he is able to somehow tell that it’s time. I have a radio, but I never have time for it. I don’t have a phone. It’s my son who reminds me.”

Female, Perfect Adherer, Age 34

- Interviewer: “Besides the phone and the radio; what else reminds you to swallow your medicine?”
- Respondent: “My children. When it’s time, they tell me ‘Mum, its time.’”
- Interviewer: “How did your children know that you swallow medicine?”
- Respondent: “I explained to them. I told those who are older; I told them the truth and showed them the pills. That time, I had not yet bought a phone; so, they would remind me always. They have a name for my medicine; they call it ‘ground nuts [a peanut common in the region].’ So, they always say that, ‘Mum, it’s time to swallow your ground nuts.’ Even if the radio is off and maybe my phone has no battery, they will come and ask ‘Mum, don’t you think it’s time for your ground nuts?’”

Female, Perfect Adherer, Age 42

Children typically become involved in adherence when asked to do so by a parent, as in the examples above. Help can also be requested from other family members to create a family network of adherence support. Creating a family adherence support network presupposes disclosure of HIV-positive status but changes its meaning. Rather than a threat, disclosure for activation of family support becomes a means to a desired end. Making adherence into “a family affair” through disclosure is a culturally congruent adherence strategy that makes effective use of available social resources. One woman described how she accomplished this:

It’s not common to forget [a dose], because when I got this medicine, I reached home and showed it to everyone, including the children, and told them the time I am supposed to swallow it. Even when I am at my mother-in-law’s place, you find her asking me whether I remembered to swallow the medicine. Even the children remind me. And when my husband is around, he also reminds me. This is what has helped me swallow my medicine well. Because when you keep it a secret and you hide it from other people, you will forget and there won’t be anybody to remind you. It’s important that other people are in the know so they can remind you.

Female, Low Adherer, Age 30

#### Relationship Support as Adherence Support for Serodiscordant Couples

Authors of a recent ethnographic analysis explaining why marriage and childbearing, as life priorities, may interfere with HIV treatment in a West African society characterized marital relationships as “collective projects in which families and communities are highly invested” [78]. In daily life, this means in-laws, friends, and neighbors may be expected to play an active role in supporting married couples and keeping their unions intact. Evidence of this was apparent in our qualitative data, which contained stories of one or another spouse seeking refuge in a neighbor’s home to “cool off” following an argument, and accounts of family interventions aimed at reconciling couples living separately due to discord in the relationship. The following examples illustrate:

When [I saw] that he [husband] was infected with HIV, I spent several months without having sex with him. I packed my bags and went to my parents’ home. I spent three months there. Then some elderly man in my husband’s neighborhood came and picked me to go back to him. I accepted. But when I reached there, he again wanted me to have sex with him. I refused totally and packed my bags again and went back to my parents. This time his brothers on his paternal uncle’s side came. They asked him the reason why we were separating. Then they concluded that since I was the only known wife, I should be left to be at home and raise my children but he gives me my peace. That is when I again went back to him. They asked him whether he agreed with the decision and he accepted. So I went back.

Female, Low Adherer, Age 39

I was going through his things and found some medicine and his medical forms. I asked him to explain what they were. He instead quarreled, asking why I went through his things without his knowledge. I decided to go home to my parents. He didn’t want me to leave but I insisted. [Later] he followed me to my home. My parents were present. I told them the reason I left was because I found my husband with pills in his bag and he told me he is HIV-positive. I told them I didn’t want to go back to his home. My parents gave us advice. They said, ‘haven’t you seen people who are positive and are living together?’ They told us to go to the hospital and seek guidance from health workers. Since the time we joined the study, we have not quarreled at all.

Female, Perfect Adherer, Age 27

In addition to family, friends and neighbors, the interviews point to other existing resources in local communities available for relationship support. These include a range of formal organizations (e.g. churches, NGOs), as well as individuals seen as community leaders (e.g., elders, tribal heads). Couples do not hesitate to draw on these resources in working to resolve threats to their relationship.

After some time, I told him, ‘if you do not want to test with me, remain alone and I also will remain alone.’ We separated. Good enough. The person who tested me is my friend, and he started advising me. …He insisted if we both went to him, he would teach us how to use a condom. Then my husband said, ‘I cannot use a condom on my wife.’ I told him I cannot accept unprotected sex. I said, ‘if this is [how it is], let us remain separate.’ So we separated our beds. Then, because we are legally married, he went and reported me to church leaders. They sat us down and talked to us about our issues. Later we went to [health center in a nearby town]. They also educated him. They told him if he does not accept, he should allow me to go back to my parents’ home. That is when he accepted and started using condoms. From that time we reconciled and became one.

Female, Perfect Adherer, Age 48

- Respondent: “He [husband] has said that the very few times I agree to have sex with him do not satisfy him. But if he was using condoms very well, I wouldn’t be having any problem. When we have sex [infrequently], they have not broken. But when the frequency of sex increases, the condoms break. …Sometimes we spend a week apart because he has chased me from home [over this]. I spend nights at different people’s homes.”
- Interviewer: “How helpful have these people been in resolving your disagreements?”
- Respondent: “They try to arbitrate but we disagree afresh. Actually, there is no place we haven’t been to [to seek help]. We started by going to the counselors at TASO [The AIDS Service Organization, Ugandan NGO]. We went to the probation office at the district. We went to the police. It is only the family members who have not met. This is the remaining option.”

Female, Low Adherer, Age 29

- Interviewer: “How did your discordant status affect how you get along with your husband in the relationship?
- Respondent: “He first wanted to always sleep with me without a condom. We feuded over this and I ended up separating from him and went back to my parents for some time. He followed me home and I disclosed to my parents what the issue was. They called a meeting of counselors and elders in the village, who gave him advice. He heeded their advice, and we agreed to use condom, which I always get from the health center, nearby.”

Female, Perfect Adherer, Age 36

Our qualitative data point to relationship partners as direct sources of adherence support to HIV-uninfected individuals taking PrEP or placebo as part of this study. If partners provide direct support, then efforts to help serodiscordant couples repair their relationships and live together without discord represent indirect resources for support of adherence already existing in communities.

## Discussion

Good adherence will be central to success in using ARVs to prevent HIV transmission and curb the epidemic. Adherence is enhanced through consistent adherence support. In Africa, professional resources for providing such support may be scarce, but informal, lay resources are likely to be both plentiful and effective. Through qualitative data, this paper offers a glimpse of what family and community-based support for PrEP adherence from spouses, other family members, and the larger community might look like for African serodiscordant couples who choose to disclose their serodiscordant status.

This paper documents the availability of social resources to support couples in adherence to ARV-based prevention. The next step will be to develop strategies and systems for deploying these resources. At least two general approaches present themselves to start. Ways of integrating laypersons into activities of clinical care—working alongside professionals in clinics as educators or counselors—need to be identified. At the same time, these resources can serve as building blocks for the construction of a community-based network that might support adherence, facilitate disclosure, combat stigma, help couples negotiate relationship problems, and educate the public about the use of ARVs for prevention. Existing community-based support organizations in which HIV-affected families are already active or have a stake might provide good starting points. Examples include church-sponsored couples’ education and support groups, civic groups active in supporting HIV-prevention activities (e.g. testing), and/or groups organized for economic self-help.

ART is a lifetime commitment, but use of PrEP by African serodiscordant couples will likely be time-limited. In East and West African Demonstration Projects, couples are being offered PrEP as a “bridge” to initiation of ART [22, 79]. In the “bridging strategy” of PrEP delivery, HIV-uninfected partners in serodiscordant couples are offered PrEP until the infected partner begins ART and is virally suppressed, at which point PrEP is discontinued. It may also be that couples will rely on PrEP when other forms of protection are not feasible (as when trying to conceive) or available. The time-limited nature of PrEP use means the need for adherence support from lay social resources will also be time-limited.

Adherence to PrEP may be more variable and more challenging than adherence to antiretroviral treatment, as the motivating effect of dramatic health gains after starting ART will be lacking. Data on PrEP adherence in demonstration projects are just beginning to appear. An early report on adherence to daily oral PrEP by HIV-uninfected partners in serodiscordant couples taking part in the Partners Demonstration Project in Kenya and Uganda shows both high adherence and frequent (unintentional) gaps and (intentional) breaks [80]. Understanding the reasons behind gaps and breaks, the associated risk of HIV exposure, and adherence levels will be central to research on PrEP adherence, given the likely variability in the ways it will be used.

Purposeful sampling intentionally represents varying perspectives on a topic of study through the inclusion of participant groups with contrasting experiences. In this sample, varying perspectives are represented through inclusion of low and perfect adherers. The sample was not constructed to identify differences among constituent subgroups; thus our analysis of group differences is limited. Qualitatively, we determined generally that identified categories of adherence support applied to both low and perfect adherers. Illustrative examples from both groups are included in the descriptions of each category as confirmation of this.

We have argued here for the importance of partners as sources of adherence support. Accordingly, discord in the relationship can detract from adherence success. A partner leaving home in anger after an argument may forget to take pills along. Tension and fighting with a spouse can undermine adherence motivation when typically the very reason for accepting ARVs is to preserve the relationship. Adherence support provided by a partner and/or by being in a partnered relationship is likely enhanced when the relationship is stable.

The following limitations of this analysis should be pointed out. The data were collected in a research setting in which adherence was both investigated and consistently reinforced. This may have resulted in greater involvement in adherence by family members and friends than might be typical outside the context of research. At the same time, the qualitative study was not designed and did not set out to elicit data on lay social resources for adherence support. The clear evidence of social involvement in adherence that emerged from the interview data suggests both that the phenomenon is real and that more could have been learned about a broader array of sources of support in the community, had this been a study focus. Nevertheless, the lack of initial focus on lay social resources for adherence support means some categories, notably the *Relationship Support as Adherence Support* category, could not be fully developed.

Data for this qualitative study were collected at a single site in one country only, limiting generalizability. Most qualitative research, this study included, does not aim for generalizability but rather at the delineation and interpretation of concepts. Qualitatively, limiting data collection to a single site in a single country means variation across communities and/or cultural settings cannot be addressed.

## Conclusion

Effectiveness of ARVs for prevention of HIV transmission depends on adherence success. Support from professionals, peers, family members and friends has been central to the excellent adherence achieved by individuals in Africa taking ART for HIV treatment.

ART scale-up has brought increasing involvement of laypersons in activities of care as a means of alleviating health care personnel shortages. Now, with evidence of the efficacy of TDF and FTC/TDF as PrEP, pressures on overburdened health systems will once again intensify. As PrEP is implemented in demonstration projects and routine health care settings, it is important to identify acceptable and effective strategies to optimize adherence and reduce burden on clinic staff. These qualitative data suggest laypersons—family members, peers, members of the larger community—constitute a promising resource for PrEP adherence support that merits further investigation.
